# Recent Update on the Molecular Mechanisms of Gonadal Steroids Action in Adipose Tissue

**DOI:** 10.3390/ijms22105226

**Published:** 2021-05-14

**Authors:** Agata Wawrzkiewicz-Jałowiecka, Anna Lalik, Graça Soveral

**Affiliations:** 1 Department of Physical Chemistry and Technology of Polymers, Silesian University of Technology, 44-100 Gliwice, Poland; 2 Department of Systems Biology and Engineering, Silesian University of Technology, Akademicka 16, 44-100 Gliwice, Poland; anna.lalik@polsl.pl; 3 Biotechnology Center, Silesian University of Technology, B. Krzywoustego 8, 44-100 Gliwice, Poland; 4 Faculty of Pharmacy, Research Institute for Medicines (iMed.ULisboa), Universidade de Lisboa, 1649-003 Lisboa, Portugal; gsoveral@ff.ulisboa.pt

**Keywords:** sex hormones, adipose tissue, metabolic disorders, insulin sensitivity, lipid metabolism, adipokines, microRNA, microRNA-oriented therapy, polycystic ovary syndrome, aquaporins

## Abstract

The gonadal steroids, including androgens, estrogens and progestogens, are involved in the control of body fat distribution in humans. Nevertheless, not only the size and localization of the fat depots depend on the sex steroids levels, but they can also highly affect the functioning of adipose tissue. Namely, the gonadocorticoids can directly influence insulin signaling, lipid metabolism, fatty acid uptake and adipokine production. They may also alter energy balance and glucose homeostasis in adipocytes in an indirect way, e.g., by changing the expression level of aquaglyceroporins. This work presents the recent advances in understanding the molecular mechanism of how the gonadal steroids influence the functioning of adipose tissue leading to a set of detrimental metabolic consequences. Special attention is given here to highlighting the sexual dimorphism of adipocyte functioning in terms of health and disease. Particularly, we discuss the molecular background of metabolic disturbances occurring in consequence of hormonal imbalance which is characteristic of some common endocrinopathies such as the polycystic ovary syndrome. From this perspective, we highlight the potential drug targets and the active substances which can be used in personalized sex-specific management of metabolic diseases, in accord with the patient’s hormonal status.

## 1. Introduction

### 1.1. Adipose Tissue Function

Adipose tissue plays an important physiological role as a central metabolic organ in the regulation of whole-body energy homeostasis in mammals and some non-mammalian animal species [1,2]. The white adipose tissue (WAT) functions as the main energy reservoir of the body, where the energy is stored in the form of triglycerides. In turn, the brown adipose tissue (BAT) accumulates lipids for cold-induced adaptive thermogenesis.

The adipose tissue is also recognized as a major multitasking endocrine organ due to its ability to produce and secrete hormones, cytokines and microRNAs, as well as a wide range of proteins of multiple functions including immune-related proteins, complement-related proteins or the proteins involved in lipid metabolism or transport [3,4]. As a consequence, the dysfunction of adipose tissue frequently leads to obesity and other metabolic abnormalities [5], as well as other health problems such as non-alcoholic fatty liver disease (NAFLD) [6,7,8,9,10]. Improper adipose tissue functioning affects even the relatively remote organs and tissues, leading to many detrimental health consequences. Adipose tissue impairment is a notable factor in cardiovascular diseases (mainly via inducing obesity-related hypertension) [11,12], has deleterious effects on microvascular and macrovascular functions [13] and promotes thrombosis [14]. Adipocytes’ malfunction affects secretion of a wide range of bioactive molecules: adipocytokines, microRNAs and inorganic compounds such as hydrogen sulfide ($H_{2}S$), which directly influence the functioning of the cardiovascular system [15,16,17,18,19,20]), evoke inflammatory illnesses (e.g., inflammatory bowel disease [21] and Crohn’s disease [22]) or have other long-range consequences, for example osteoarthritis [23,24,25].

**Figure 1:**
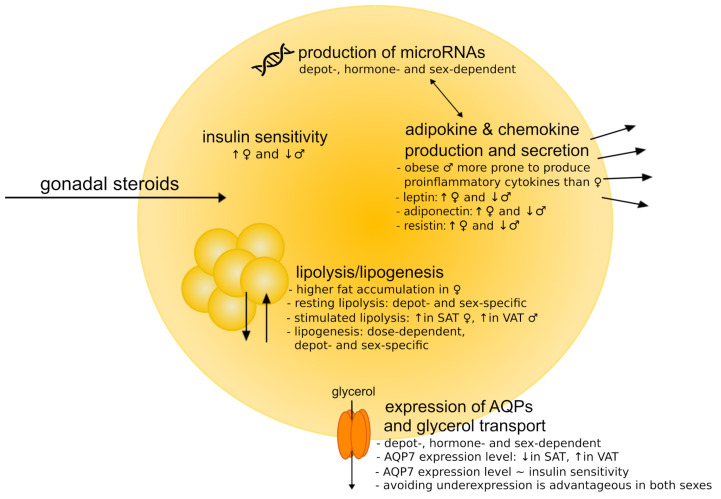
Schematic representation of the most important processes of adipocyte functioning affected by gonadal steroids and the main sex-differences. Sex hormones influence the expression levels of microRNAs, lipolysis and lipogenesis, insulin sensitivity and endocrine function of adipocytes (e.g., adipokine production). One of the most prone connecting links between sex-hormonal imbalance and the disruption of adipose tissue functions is the expression level of aquaporins (AQPs) (in particular, aquaglyceroporins) and, consequently, the effectiveness of the glycerol efflux from adipocyte.

The adipose tissues are recognized as significant sites for transformation of sex steroid hormones and their action, as recently summarized in [26,27]. In turn, gonadal steroids are important factors in the determination of fat distribution and accumulation [28,29,30,31,32]. Moreover, sex steroid hormones clearly exert detrimental effects on adipocytes functioning, including lipolysis and lipogenesis, insulin sensitivity and its endocrine role (e.g., adipokine production and regulation of microRNAs expression) [26,27,33], as briefly summarized in Figure 1.

The primal factor that controls the function and metabolism of adipocytes (as all other cell types) is the level of gene expression, which can be post-transcriptionally regulated by microRNA. In turn, the expression of microRNAs is controlled by biochemical agents, including gonadal steroids and adipokines [34,35,36,37,38].

### 1.2. Gonadal Steroids

**Figure 2:**
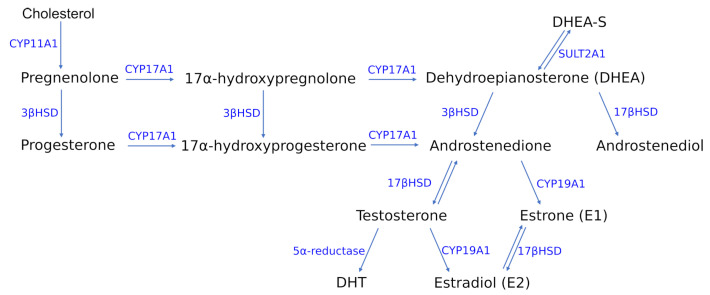
Adipose tissue is an important side for sex hormone interconversions. This figure summarizes the most prominent reactions of this kind. All sex hormones can be synthesized de novo from cholesterol in presence of active steroid hormones. However, more frequently in adipose tissue, the hormones are taken up from plasma and further transformed into other ones. The direction and efficiency within the steroid biosynthetic pathways in adipose tissue depends on the relative expression and activity of steroidogenic enzymes, which are gender-, age- and depot-specific. Abbreviations: CYP11A1, cholesterol side-chain cleavage enzyme; CYP17A1, steroid 17α-monooxygenase; 3β-HSD, 3β-hydroxysteroid dehydrogenase; 17β-HSD, 17β-hydroxysteroid dehydrogenase; SULT2A1, dehydroepiandrosterone sulfotransferase; DHT, dihydrotestosterone.

The group of gonadocorticoids, commonly known as sex hormones or sex steroids, is constituted by progestogens, androgens and estrogens. The first representatives of the sex hormones are progestogens, with progesterone as the major and most physiologically important in the human body. Progesterone is produced from cholesterol through a series of reactions and intermediates (Figure 2). In the initial step, cholesterol is converted into pregnenolone, which serves as the precursor to the progesterone and 17α-hydroxyprogesterone. Along with another steroid, 17α-hydroxypregnenolone, these progestogens are the precursors of all other endogenous steroids [39,40] (Figure 2). Progesterone is mainly recognized as the hormone required for maintaining pregnancy.

Among the most potent androgens in human organism are testosterone (T), dihydrotestosterone (DHT), androstenedione, dehydroepiandrosterone (DHEA) and dehydroepiandrosterone sulfate (DHEA-S) [41]. Within the estrogenes the most prominent physiological role in the human body is played by the 17β estradiol (E2), followed by estrone (E1) and estriol (E3) [42]. E2 is produced from androgens through the pathway involving formation of androstenedione, which is subsequently converted by aromatase (CYP19A1) into estrone and then transformed into estradiol (Figure 2). It can also be synthesized through the second pathway that is based on the interconversion of steroids from androstenedione into testosterone in the presence of 17β-hydroxysteroid dehydrogenase (17β-HSD), which is then converted into estradiol [40] (Figure 2).

### 1.3. Perspectives

In this review, the effects of androgens, progestogens and estrogens on adipose tissue functioning are examined considering the recent advances addressing the molecular aspects of that phenomenon. The direct biochemical mechanisms of gonadal steroids action on the adipocyte functioning are here discussed as well as the most notable possibilities of their indirect modulation. In addition, considering the involvement of aquaporins (AQPs), particularly aquaglyceroporins, in adipocyte biology [43] (Figure 1), we discuss the indirect effects of gonadal steroids on energy metabolism via modulation of aquaglyceroporins. It may give a mechanistic substantiation of the observed impairment of adipocyte functioning in terms of sex-hormonal imbalance that has not been broadly discussed in the context of gonadal steroids action in adipose tissue, hitherto. In this work, the hormonal imbalance pathogenic impact is also analyzed taking into consideration the possible post-transcriptional regulation of gene expression via microRNA.

The identification of at-risk populations taking into account the gender differences in obesity and metabolic dysfunctions, e.g., the worldwide female predominance in obesity or the male prevalence in the total cases of type 2 diabetes (T2D) [44], will foster new therapeutic procedures for diseases leading to sex steroids’ imbalance, which are often gathered by metabolic disturbances, including endocrinopathies (e.g., polycystic ovary syndrome (PCOS) [45,46] and hypogonadism in men [47,48]) or autosomal recessive disorders (e.g., congenital adrenal hyperplasia [49,50]). Other health conditions, such as the case of the overweight men with visceral adipose tissue accumulation who are at relatively high risk for low androgen levels and developing T2D [51,52,53] or the decreased estrogen and progesterone levels accompanied by increase in overall adiposity due to visceral adipose tissue accumulation in menopausal women, clearly underscore the importance of sex steroid hormones in proper functioning of adipose tissue [54]. Moreover, this work highlights the sexual dimorphism in the regulation of energetic, glucose and lipid homeostasis, shedding new light on the need of development personalized sex-specific approaches in therapies against metabolic diseases.

The design of efficient therapies to alleviate metabolic abnormalities taking into account the sex dimorphism programmed by the hormonal disparities in men and women should be preceded by unraveling the mechanism of their pathogenesis at the molecular level in a step-by-step manner. From such a perspective, we summarize here the most promising scientific developments in the search of novel drug targets, as well as active substances, which can act on the initial causes of the metabolic disorders at a molecular level. The potential sex-specificity of their therapeutic efficiency is also discussed.

## 2. Effects of Gonadal Steroids on Body Fat Distribution in Humans and Adipocyte Morphology

**Figure 3:**
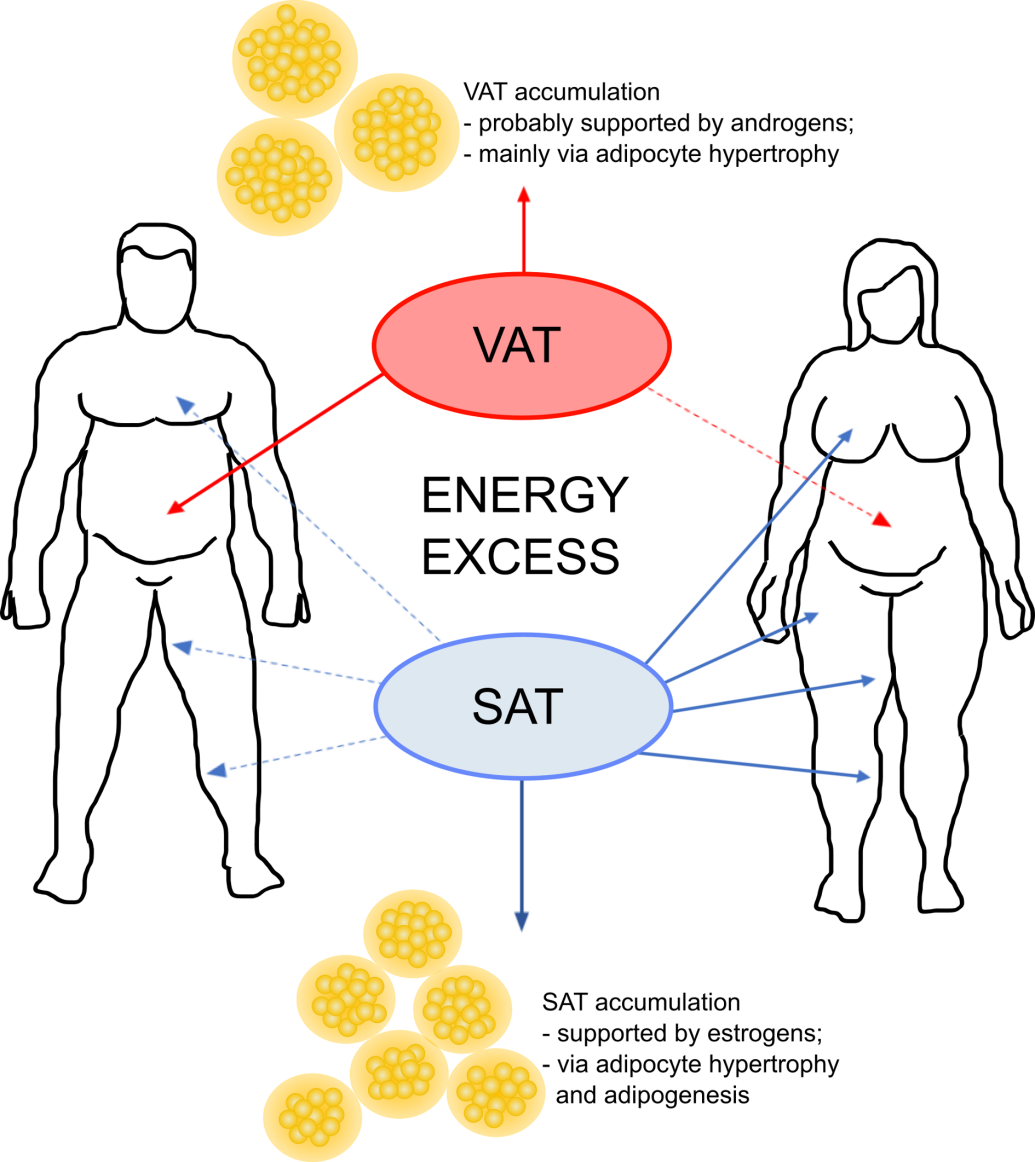
Schematic representation of sex-dependent differences in the fat deposition patterns. The visceral adipose tissue (VAT) is located around the abdominal organs and is the main type of fat depots in men. On the contrary, women distribute fat mostly in the form of subcutaneous fat. In general, increased visceral mass predominantly results from adipocyte hypertrophy, whereas subcutaneous fat (SAT) grows through both hypertrophy and hyperplasia (with prevailing hyperplastic mechanism) [77,78]. Considering sex-dependent differences in growth of gonadal fat depots, it is observed that, in response to energy excess, male gonadal depot grows mainly through hypertrophy, while female gonadal depots expand both hypertrophy and hyperplasia [79].

One of the most evident impact of the gonadal steroids on the body refers to the adipose tissue distribution [31]. There are significant sex differences in body composition [31,44,55] (Figure 3), where women have a higher percentage body fat than men and accumulate fat mainly in the form of subcutaneous adipose tissue (SAT), creating a “gynecoid” type of distribution (in gluteal-femoral depots) [56]. In turn, men tend to accumulate their fat as visceral adipose tissue (VAT) around the abdominal organs [57]. The abdominal VAT deposition correlates with an increased susceptibility for cardiometabolic complications [58,59]. On the contrary, the gluteal-femoral adipose tissue distribution plays a protective role against the adverse health effects of metabolic diseases [60,61].

Sex-specific fat distribution is influenced by several factors, including genetical factors as well as hormonal and diet status [31,62,63]. The most prominent effect is, however, exerted by the hormonal bias, which is clearly visible by comparison of premenopausal and postmenopausal women. The reduction in the levels of estrogen after menopause results in increased fat storage in abdominal depots [64,65,66].

The effects of sex steroid hormones on adipocyte differentiation and morphology, and consequently on fat deposition, are mediated by the presence of their receptors including α-ER and β-ER estrogen receptors, α-PR and β-PR (progesterone receptors) and the α-AR and β-AR adrenergic receptors [28,67,68]. These receptors are expressed in sex- and depot-dependent manner in preadipocytes and adipocytes within the human body [69].

As suggested by the analysis of a mouse model, the impairment in estrogen signaling evoked by the knockout of ERα results in obesity, insulin resistance and diabetes regardless of gender [70,71,72,73]. According to the literature [28], the AR gene expression is affected by the gonadal steroids in both preadipocytes and adipocytes, which is important in shaping the sex-related differences in adipose tissue regional distribution. It turns out that $\alpha_{2A}$-AR is the prevailing AR subtype expressed in preadipocytes, whereas, in mature adipocytes, the $\beta_{3}$-AR is dominant [28].

Although the sex hormone receptors are expressed in preadipocytes as well as in adipocytes of VAT and SAT depots in human body, some sex-dependent differences of expression level occur. The subcutaneous adipose tissue has higher concentrations of ERs and PRs compared to ARs in females, and E2 downregulates AR expression in SAT [70,74,75]. In contrast, visceral adipose tissue has a higher concentration of ARs [74,76]. These differences in expression level of sex steroid receptors may highly influence the differentiation and morphology of adipocytes [27].

In terms of energy excess, the adipose tissue expands by increasing the number (hyperplasia) and/or size (hypertrophy) of adipocytes [80,81]. Hyperplastic growth is considered to be healthier in opposition to the hypertrophic expansion, which leads to adipocyte death, lipotoxicity, insulin resistance and high inflammation [82,83,84,85]. In response to energy excess, the typical male VAT depots expand mainly through hypertrophy [82], while female gonadal and subcutaneous depots grow through both hypertrophy and hyperplasia. This has reasonable grounds considering the hormonal bias. Namely, proliferation of preadipocytes is upregulated by estrogens with greater effects in preadipocytes isolated from SAT vs. VAT and females vs. males [79]. In turn, androgens hamper adipogenesis in in vitro and in vivo trials and the effects are greater in preadipocytes from VAT vs. SAT [79,86,87,88,89,90].

Considering the role of progesterone in body fat accumulation, it was proposed that this sex hormone might be responsible for gynoid fat distribution in premenopausal women in an indirect mechanism. It may attenuate the effects of cortisol in WAT via glucocorticoid receptor, therefore, it hampers cortisol-related central fat accumulation [91]. The analysis of progesterone action on murine and human preadipocytes differentiation gave inconsistent results. It was found that progesterone stimulated adipogenesis in the 3T3-L1 adipose cell line [92], however it was without effect on cultured human preadipocytes [93].

An important contributor to sex bias in adipose tissue distribution and adipocyte size may be the rate of direct fatty acid uptake by tissues. Direct fatty acid uptake is higher in the abdominal depot in men and in the gluteal-femoral depot in women [94]. Another potential factor that contributes to sex differences in adipose tissue expansion is nourishment [27]. In a mouse model, the number of adipocyte precursor cells in gonadal or subcutaneous fat is highly dependent on diet [95].

## 3. The Direct Influence of Sex Steroids on Adipocyte Functionality

This section summarizes the molecular mechanisms of the direct effects of gonadocorticoids on versatile aspects of adipose tissue functioning. Sex hormones frequently act on adipocytes in a sex-dimorphic, dose-dependent and depot-specific manner.

### 3.1. Lipolysis and Lipogenesis

The processes of lipolysis and lipogenesis within adipose tissue control the energy release and energy storage in the body. During lipolysis triglycerides are hydrolyzed into glycerol and free fatty acids (FFA). There are significant differences between men and women in basal lipolysis rates in terms of resting energy expenditure which occur in a depot-specific manner [75]. There are also sex-differences in stimulated lipolysis, being greater in women’s abdominal SAT and in men’s VAT [31,75].

Consistent with an inhibitory effect on adipogenesis, androgens have been shown to increase lipolysis, and consequently support adipocyte hypertrophy [68,96]. In a rat model, isoproterenol and noradrenaline-stimulated lipolysis were increased by T (but not DHT) in male preadipocytes [97]. An additional stimulating effect is exerted by DHEA via upregulation of adipose triglyceride lipase (ATGL) and hormone-sensitive lipase (HSL) mRNA [98]. In humans, lipolysis is enhanced by androgens in a dose-dependent and depot-specific manner. The in vitro studies have found that testosterone and dehydroepiandrosterone stimulate norepinephrine-stimulated lipolysis and depresses lipoprotein lipase activity (LPL) in adipose cells [99].

The results presented in [100,101] indicate that castrated rodents display reduced basal and catecholamine-stimulated lipolysis, but introduction of the testosterone supplementation allowed to fully normalize their lipolytic activity. Another study showed that androgens can downregulate hormone-sensitive lipase (HSL) and $\beta_{2}$-AR expression, and consequently reduce catecholamine-stimulated lipolysis, particularly in subcutaneous adipocytes [102]. The investigation of the effects of dihydrotestosterone on differentiation and proliferation of human mesenchymal stem cells and preadipocytes by Gupta et al. [87] showed that DHT decreased differentiation of fat cell precursors, increased lipolysis and reduced lipid accumulation. The androgenic effects on lipolysis are believed to be mediated by the AR since flutamide (being the AR antagonist) significantly abolished these effects [103]. However, other additional mediators are also considered, e.g., hormone-sensitive lipase and adenylate cyclase [97,100,101,104].

Analyzing the effects of estrogens on lipid storing and mobilization, it is reported that E2 allows to suppress lipogenesis and lipogenic gene expression and promote both basal and catecholamine-induced lipolysis [26]. As mentioned above, lipolysis is controlled to a large degree by hormone receptors. The effects of estradiol on lipogenesis are mediated by α-ER receptor activation, which reduces LPL activity (fundamental for fat uptake into the adipocytes) and increases β-AR and $\alpha_{2A}$-adrenergic receptors in SAT [105]. The examination of women with low estrogen levels also indicated reduced LPL activity when the hypogonadal women obtained estrogen replacement therapy [106]. In contrast, estradiol exerts no effects on $\alpha_{2A}$-adrenergic receptors mRNA expression in adipocytes from the visceral fat depots [105]. Thus, the effects of estrogens on lipolysis are depot-specific, facilitating the typical female subcutaneous fat accumulation.

Estradiol also exerts additional effects on lipid metabolism. Namely, the E2 downregulates adipogenic peroxisome proliferator-activated receptor γ (PPARγ) expression and the key lipolytic genes including the ones encoding stearoyl-CoA desaturase 1 (SCD1), fatty acid synthase (FAS) and acetyl-CoA carboxylase 1 (ACC1) [68,107]. E2 also enables increasing muscle oxidative capacity [54,108] through the regulation of acyl-CoA oxidase and uncoupling proteins (UCP2-UCP3), which enhances fatty acid uptake without lipid accumulation.

The effects of progestogens, especially progesterone, on lipid metabolism are not as broadly described as the influence of other corticosteroids. There are contradictory reports of the role of progesterone in lipogenesis and lipolysis, with the dominating view that this hormone supports lipogenesis and hampers lipolysis. As indicated in [109], in diabetic rats, progesterone stimulates lipogenesis in adipose tissue without any increase in food intake or serum insulin concentrations. Moreover, the results presented in the work of Stelmańska et al. [110] indicate that the elevated blood progesterone concentration is associated with significant increase in expression of lipogenic enzyme genes (Srebf1 and S14 genes) in inguinal WAT of female rats. Another rodent study demonstrated that progesterone acts in a sexually-dimorphic way in rats [111]. In female rats, progesterone induced downregulation of hormone sensitive lipase and upregulation of G0/G1 switch 2 (G0s2) genes expression in inguinal white adipose tissue, which was reflected by lowered rate of stimulated lipolysis. In turn, in male WAT, progesterone has no effect on the expression of aforementioned genes. The last inference is in agreement with in vivo studies [112,113] that indicated that progesterone has no influence on lipid metabolism in male rats’ adipose tissue. The former studies indicated that progesterone inhibits lipogenesis [114] and enhances lipolysis [115], whereas another study indicated that progesterone stimulates lipogenesis [116].

### 3.2. Insulin Sensitivity

Men are less sensitive to insulin than BMI- and age-matched women [26,117,118,119]. Obesity is one of the main risk factors for T2D. Although obesity is more frequently diagnosed in women than men, T2D occurs with increased prevalence in men [44]. Considering the molecular effects of gonadal steroids on insulin sensitivity, one has to realize that glucose-insulin homeostasis is systemically regulated in both sexes through hormone receptors [26,70].

The regulatory role of α-ER is mediated by modulation of the tyrosine phosphorylation of insulin receptor substrate 1 (IRS-1) protein [120]. Estradiol is able to activate adenosine monophosphate-activated protein kinase through ER, and consequently enhance the activity of protein kinase B (AKT) via 5${}^{\prime }$ AMP-activated protein kinase (AMPK), according to the in vitro studies presented in [121]. The role of androgens in glucose homeostasis is highly dimorphic in men and women, which is evident by the liability of hyperandrogenemic women (e.g., in the case of PCOS) and hypoandrogenemic males (suffering hypogonadism) to insulin resistance and obesity [122,123]. Administration of testosterone to differentiated, subcutaneous preadipocytes from lean women causes insulin resistance via insulin-stimulated phosphorylation of protein kinase C ζ (PKCζ), which initiates the translocation of glucose into the cell via glucose transporter type 4 (GLUT4) [26,124]. In turn, in men, the insulin sensitivity can be modulated by 5α-reductase (an enzyme involved in interconversion of T to DHT (Figure 2)) [125,126].

The studies on a mouse model of PCOS indicated that the low-dose DHT causes lowering of the components of insulin signaling (e.g., GLUTs) in energy storage tissues but a simultaneous increase in the levels of insulin signaling components in reproductive tissues [127]. Another androgen, DHEA, is reported to increase glucose-stimulated insulin secretion in animal models [26,128,129]. Nevertheless, the data obtained from human studies significantly vary [129]. The general opinion states, however, that DHEA should evoke insulin sensitization and counteract obesity through downregulation of 11β-hydroxysteroid dehydrogenase type 1 (11β-HSD1). Thus, DHEA might be considered as a promising target for the treatment of obesity and diabetes [130,131,132].

### 3.3. Endocrine Function of Adipocytes

In recent years, metabolic diseases including obesity, metabolic syndrome and T2D have become even more common. They result in the excess adiposity, which sustains a state of chronic low-grade inflammation characterized by infiltration of immune cells (mainly macrophages) into adipose tissue [133]. The infiltrated immune cells are, in turn, able to release inflammatory cytokines and chemokines [134]. Moreover, the presence of pro-inflammatory cytokines impairs insulin signaling, further leading to insulin resistance [134,135,136], among others. It can also result in endothelial dysfunction and subsequent atherosclerosis [27]. VAT, which is the predominant type of excess adipose tissue in men, has a greater number of adipose tissue resident macrophages in comparison with SAT depots, which is the predominant fat depot in women. Thus, obese men are more prone to produce pro-inflammatory cytokines than BMI-matched women, and, further, these sex-differences are also preserved in the relative number of insulin resistance cases and endothelial dysfunction morbidity. Nevertheless, the presence and functional impairment of abdominal fat are significant risk factors for sexual dysfunction in both genders [137,138].

#### 3.3.1. Leptin Production

Leptin is an endocrine hormone able to regulate immunity and energy homeostasis (by exerting an anorectic effect related to its action in the hypothalamus and, further, by enhancing lipolysis). Gonadal steroid hormones are able to determine the sexual dimorphism in serum leptin level, which is higher in women than men regardless of age [79]. The literature suggests that the estradiol increases leptin secretion from omental adipocytes in women by controlling synthesis of transcripts encoding leptin and the expression of leptin-specific receptors [79,139], but the E2 exerts no effects on men adipocytes [140]. Additionally, leptin concentrations in postmenopausal women are lower than in premenopausal women [54]. The positive influence of estrogen on leptin level in females was also confirmed in an animal model [141].

An opposite effect is exerted by androgens. Both testosterone and dihydrotestosterone decrease leptin gene expression and secretion from human adipocytes [142]. As can be inferred from the in vitro studies on 3T3-L1 murine adipocytes, DHT probably reduces the level of secreted leptin by decreasing its transcript abundance [139].

#### 3.3.2. Adiponectin Production

One of the crucial anti-inflammatory adipokines is adiponectin, which improves insulin sensitivity by suppressing hepatic glucose production and enhancing fatty acid oxidation in the liver and skeletal muscles. The plasma adiponectin concentrations are inversely correlated with the size of adipose tissue reservoir [143]. There is an evident sexual dimorphism in circulating adiponectin levels, i.e., men have lower adiponectin levels than women when matching BMI and age groups are compared [144]. This discrepancy between sexes is related to androgen levels, confirmed in cell culture models as well as in an animal model where castration increases adiponectin level that additionally could be attenuated by testosterone supplementation [144]. In line with these observations, men with hypogonadism have significantly higher total serum levels of adiponectin in comparison to healthy controls, which lowers along with the introduction of testosterone replacement therapy [79,145]. In turn, women suffering from PCOS have relatively low adiponectin levels [146,147] which makes them prone to insulin resistance.

#### 3.3.3. Resistin Production

Resistin is a hormone produced by adipocytes and contributes to obesity and T2D. In a mouse model this peptide hormone caused insulin resistance when it was exogenously supplied [148]. In the same study, administration of anti-resistin antibodies improved blood sugar and insulin action in mice with diet-induced obesity. Human studies showed that resistin is relatively high in adult women, but does not differ in boys and girls throughout the stages of puberty [149].

Some observations were made during the analysis of human endocrinopathies. Munir et al. [150] showed that serum resistin concentration was positively correlated with BMI and testosterone level in PCOS women, but not in controls. In turn, the mean serum resistin concentration was increased (of 40%) in women with PCOS. The results presented by Seow et al. [151,152] show that the mRNA resistin level in adipocytes was higher in PCOS women than in controls [147], although other reports suggest no PCOS-related effects on resistin concentration or expression [153,154]. In the case of hypogonadal men, introduction of testosterone replacement therapy does not affect circulating resistin concentrations in serum [155].

#### 3.3.4. Production of Pro-Inflammatory Adipocytokines

There are some sex-related differences in human immunity, which partially stem from the influences of different gonadal steroids on leukocyte biology but also are inclined by genes on sex chromosomes [156]. The levels of gonadal corticoids may deeply influence the adipose tissue immune cell populations via regulation of their proliferation, differentiation and apoptosis [157,158,159,160]. Further, estrogens and androgens regulate the secretion of bioactive molecules by immune cells, which are related to inflammation, endothelial functionality and insulin sensitivity in adipocytes [26,27,79,158,160,161]. The effects of estrogens and progestogens on cytokine and immunoglobulin production in different immune cell types (e.g., T lymphocytes, monocytes, B lymphocytes, granulocytes, natural killer cells and dendritic cells) were summarized by Oertelt-Prigione [160], and an analogous detailed analysis of the androgen effects on immune cells is provided in [157,159,161].

As mentioned above, sexual dimorphism in immunity manifest itself in the disparate abilities to produce inflammatory biomarkers in men and women, which include C-reactive protein (CRP), tumor necrosis factor (TNFα) and some interleukins (IL-1, IL-6 and IL-12). In addition, there are notable differences in responsibility and activity of other molecules and molecular complexes regulating the immune response to infection such as the nuclear factor kappa-light-chain-enhancer of activated B cells (NF-κB) [137,156]. The in vitro studies from Ghisletti et al. [162] showed that the estradiol administration blocks the inflammatory response. According to the study on ovariectomized female mice, administration of estrogens significantly lowers the mRNA levels of IL-6, TNFα and CD68. In addition, estrogen prevented female mice from developing liver steatosis and from becoming resistant to insulin [54,163]. Nevertheless, chronic estradiol administration to ovariectomized mice enhances pro-inflammatory cytokine production of IL-1β, IL-6 and TNFα [164,165]. Considering the mechanism of the anti-inflammatory effects of estradiol, one of the component processes is that E2 prevents transcription of genes encoding pro-inflammatory mediators (inhibiting intracellular transport of p65, which is a member of NF-κB family, to the nucleus) [162,166]. This activity is selectively mediated by α-ER receptor [156,162,167].

Most innate immune inflammatory cytokines (IL-6, IL-8, TNFα), are inhibited in women by periovulatory dosages of estrogen. The studies on postmenopausal women show that low levels of estradiol can augment inflammatory mediators, which could explain the pro-inflammatory states in this group of women [168].

Considering the role of androgen in the immune system, androgens administration in hypogonadal men may reduce systemic inflammation. Testosterone is reported to have immunosuppressive and anti-inflammatory functions. In fact, this hormone was found to lower IL-6 and TNF-α levels through inhibition of the NF-κB pro-inflammatory pathway, analogously to estrogen [169]. Consequently, testosterone replacement in hypogonadal men can give rise to increased serum levels of IL-10 and reduced TNFα and IL-1β [170].

### 3.4. Sex-Related Discrepancies in Immunological Responses

The immunological responses mediated by adipose tissue functioning present some sex-related discrepancies. Cytokine secretion by the peripheral blood mononuclear cells (PBMCs) occurs in a gender-dependent manner, which coincides with elevated estrogen levels [171]. The PBMCs from men produce more pro-inflammatory TNFα and less protective IL-10 than PBMCs from women, which may explain a better outcome of diseases such as sepsis in females.

Susceptibility to viral infections as well as their severity are higher in men than in women, which was broadly discussed due to the current COVID-19 crisis [156,172,173,174,175]. This variability could be partly explained by the sex-differences in the activity of the Toll-Like Receptors (TLR) which are involved in the virus recognition. Higher male liability to viral infection can be in part attributable to higher cytokine secretion by the PBMCs leading to enhanced TLR9 activation and increased IL-10 production, which is positively correlated with androgen concentrations [172].

The results from Channappanavar et al. [173] based on a mouse model of SARS-CoV infection matched the appropriate epidemiological data from SARS outbreaks and indicated a notable male bias in disease susceptibility. It also demonstrated a protective effect for estrogen receptor signaling in mice infected with SARS-CoV (ovariectomy or treating female mice with an estrogen receptor antagonist increased mortality). The recent data describing the profiles of SARS-Cov-2-infected patients show that the levels of several important pro-inflammatory innate immunity chemokines and cytokines (e.g., IL-8 and IL-18) are higher in male patients. However, more robust T cell activation is exhibited by women than men. A poor T cell response is negatively correlated with patients’ age and is related to worse disease outcome in male, but not female, patients [174]. From this perspective, it is possible that estrogen plays a similarly protective role against SARS-CoV-2 as in the case of SARS-CoV. Analogously, relatively high testosterone exerts an anti-inflammatory effect in men and frequently protects younger men against adverse outcomes of SARS-CoV-2 infection.

## 4. Modulation of Aquaporins by Sex Steroids as an Indirect Mechanism of Adipose Tissue Regulation

The studies on pathogenesis of metabolic diseases requires detailed analysis of the molecular mechanisms of the gonadal steroids on adipose tissue. We are convinced that in this case not only the direct biochemical pathways already broadly discussed in literature should be analyzed. As in every scientific discussion, one should also consider a global view of the research problem and point out its critical components which shape the main cause and effect relationships. One of the connecting links between sex-hormonal imbalance and the disruption of adipose tissue function is the activity of aquaporins.

As a fundamental property of life, the flow of water and small solutes across cell membranes is crucial for the correct course of physiological processes. Aquaporins (AQPs) are transmembrane proteins responsible for bidirectional transport of water and small solutes (including ammonia, CO${}_{2}$, glycerol and urea) across biological membranes in response to osmotic or solutes’ gradients [176]. Thus far, thirteen AQP isoforms have been identified (AQP0–AQP12) in human [177]. They can be divided into two main classes based on their permeability characteristics [178]. The “classical” ones are mainly selective to water (AQP0, AQP1, AQP2, AQP4, AQP5, AQP6 and AQP8). The other group is formed by aquaglyceroporins (AQP3, AQP7, AQP9 and AQP10), being able to transport small non-charged solutes such as glycerol and urea. The structure and function of the remaining AQP11 and AQP12, sometimes called “unorthodox” or “superaquaporins”, are relatively poorly understood. Moreover, among aquaporins, the group of ammoniaporins (AQP3, AQP6, AQP7, AQP8 and AQP9) is recognized for their ammonia transport ability [179,180] while peroxiporins (AQP3, AQP5, AQP8, AQP9 and AQP11) guarantee the efficient transport of H${}_{2}$O${}_{2}$ across the plasma and organelle membranes [181,182,183,184,185,186].

The members of the aquaglyceroporin subfamily exert a high impact in the crucial aspects of adipose tissue functioning, mentioned in the former sections of this work. Namely, AQP7 is considered as the main glycerol channel in adipose tissue, facilitating the release of lipolytic glycerol from adipocytes in response to the energy demand [43,187,188,189,190]. AQP7 deficiency results in reduced membrane glycerol permeability which leads to increased triglyceride accumulation inside adipocytes and adipocyte hypertrophy contributing to the onset of obesity [188,191,192,193]. AQP7 is not the sole aquaglyceroporin in adipose tissue [43]. Recently, AQP3, AQP9, AQP10 and AQP11 were additionally identified in human adipocytes and suggested as additional glycerol pathways in these cells, unveiling their role as key players in lipid balance and energy homeostasis [194]. However, AQPs are differentially expressed in the two types of fat depot. Whereas visceral fat show increased AQP3 and AQP7 levels which can be correlated with increased lipolysis and glycerol release, in subcutaneous fat, AQP7 expression is lower, favoring fat accumulation and adipocyte hypertrophy [43,187]. In addition, gender differences in glycerol metabolism have also been reported in healthy and obese humans, with women showing higher fasting circulating glycerol levels and higher AQP7 expression than men in both subcutaneous and visceral adipose tissue probably due to hormonal regulation of adipose and liver aquaglyceroporins [195,196]. The sexual dimorphism in fat distribution and the gender-specific AQP7 levels may contribute to the lower prevalence of insulin resistance and metabolic disorders found in premenopausal women compared to men [196].

Aquaglyceroporins involvement in adipose tissue dysfunction and lipotoxicity in liver and muscle highlights their potential as therapeutic targets for the metabolic syndrome [197]. The literature reports indicate a close relationship between AQP7 and insulin sensitivity [188,198]. AQP7 is downregulated in terms of insulin resistance according to the in vitro studies on mice adipocytes where insulin resistance was induced by dexamethasone or TNFα [198]. Furthermore, overexpression of AQP7 contributes to improve sensitivity to insulin [198]. According to the results obtained in mice [199], the suppression of AQP7 expression by insulin proceeds through an insulin negative response element (IRE) located on the promoter region of the Aqp7 gene and is mediated by the phosphatidylinositol 3-kinase (PI3K) pathway. The human studies showed that the AQP7 gene was also negatively regulated by insulin via an insulin response element [200]. Moreover, the gene encoding AQP7 is localized in a chromosomal region (9 p13.3–p21.1) with reported linkage to the metabolic syndrome and to T2D [201,202].

It is also worth mentioning that peroxiporins are especially important in functioning of the immune system [203]. Due to their ability to facilitate hydrogen peroxide fluxes across biological membranes, they are tightly involved in redox balance and modulation of oxidative stress [186] and were reported to regulate immunological processes including inflammasome priming and activation [204].

Moreover, the water transport via aquaporins in immune cells enables them for shape and volume changes as well as maintaining an appropriate osmotic gradient across cell membrane, which are critical factors in cell-to-cell communication (via chemokines) and induction of changes in shape for migration, phagocytosis or antigen uptake [203]. Thus, aquaporins provide also a direct hydromechanical support for immune cells which is essential for their proper functioning.

Considering the merit of this work, it is worth emphasizing that aquaporins play an important role in female and male reproductive systems [205,206,207], and the alteration of their expression can be frequently linked with infertility [206,208], among others. Hormonal alterations are a known factor leading to reduced reproductive health and might frequently be associated with altered AQPs expression and function not only at a gonadal level, but such effects can occur systemically.

The regulation of AQPs level by estrogen is relatively well documented in literature. This steroid hormone can regulate AQP expression in the female reproductive system (uterus, vagina, ovary, cervix and placenta) [209,210], as well as in the men reproductive system (efferent ducts, epididymis and Sertoli cells) [206,211,212,213,214]. Rodent and human studies suggest that there is a positive correlation between aquaporin expression levels (particularly AQP1–APQ3) and the 17β-estradiol concentration in gonads and in serum in females [207]. The opposite relation between E2 and AQPs is found in men, where the levels of AQP1, AQP3 and AQP9 were broadly studied [206]. In that case, however, the downregulation of AQPs expression (AQP3 and AQP9) by E2 in men is thought to be partly restored by testosterone administration [213,214].

There are sparse data describing the direct effects of testosterone on the abundance of aquaporins within the body. To shortly sum up, testosterone is reported to increase the AQP1, AQP5 and AQP7 expression in ovariectomized rats, which may explain the observation that the T hormone is linked to a decrease in uterine fluid volume [215]. In male rats, APQ9 abundance in epididymal epithelium is modulated by testosterone [216]. In addition, the rate of ductus deferens fluid secretion under testosterone influence is mediated via the upregulation of AQP1, AQP2 and AQP9 [217]. In turn, decreased prostatic and seminal vesicle secretions during the ongoing deprivation in androgen level is related to the decrease in AQP0, AQP1, AQP4, AQP5, AQP6 and AQP8 in prostatic tissue and seminal vesicles [218].

Additionally, higher blood pressure is recorded in males as compared to females. In turn, within women, there are also changes in tendencies to hypertension between the premenopausal and postmenopausal ones, where the later are more prone to high blood pressure. There is a hypothesis that these phenomena can be explained by the testosterone-induced increase in blood pressure mediated by the changes in aquaporin expression in kidneys. The studies on a rat model confirm that hypothesis [219]. Testosterone administration causes exaggerated AQPs expression in kidneys which results in H${}_{2}$O retention and further gives rise to changes in the blood pressure [219]. Testosterone is also thought to influence brain edema via altering the osmotic fragility of astrocytes through AQP4 regulation [220].

Progesterone may also influence the aquaporins expression level, and this kind of influence plays a critical role during pregnancy. According to the results obtained in [221], progesterone upregulates the expression of AQP1 in the rat placenta and enhances abundance of AQP1 and AQP5 in the uterine tissue [222]. Progesterone exerts a similar effect in women to estrogen [207]. There is a positive correlation between progesterone level in serum and the AQP2 expression levels in endometrium [223]. Interestingly, an AQP3 functional genetic polymorphism was found associated with hypertension in women only after pregnancy [224].

What are the additional potential mechanisms of how the changes in steroid hormone levels may affect the AQPs expression within the organism (and particularly in adipose tissue)? A key to answer that question is the fact that the AQP7 expression in fat cells is sensitive to glucocorticoids, fasting–refeeding, insulin, TNFα, adrenoceptor agonists and peroxisome proliferator-activated receptor (PPAR) stimulation [192,199,225]. As summarized in the previous part of this work, most of the mentioned factors are deeply affected by the level of gonadal steroids. Thus, hormonal imbalance exert an indirect effect on aquaporins. This effect is, however, an important factor contributing to the observed metabolic complications in patients suffering abnormal sex hormone levels. For example, the excess amount of androgens in women (e.g., in PCOS) leads to the development of metabolic complications including global adiposity, adipocyte hypertrophy and its dysfunction—frequently leading to central obesity. In turn, obesity leads to genetic polymorphism (A-953G SNP) causing underexpression of AQP7 in adipocytes [226]. Furthermore, AQP7 deficiency results in increased glycerol concentration inside adipocytes and increased activity of glycerol-3-phosphate leading to preferential re-esterification of FFAs. This sequence of events enhances progressive triacylglycerol accumulation and adipocyte hypertrophy.

## 5. MicroRNAs Expressed in Adipose Tissue Are Involved in Molecular Mechanism of Metabolic Diseases

The function and metabolism of cells are tightly controlled and depend on gene expression, regulated both at the transcriptional and post-transcriptional levels. Small, noncoding RNA molecules called microRNAs play an important role in the post-transcriptional regulation of gene expression and have been shown to play an important role in all cellular processes. Deregulation in the expression of microRNAs leads to cell dysfunction and the development of disease states [227].

The biology of adipose tissue cells has been found to be regulated by microRNA [228]. In adipocytogenesis, microRNAs control all stages of cell differentiation. For example, the switch between the differentiation of mesenchymal stem cells towards adipogenic vs. osteogenic lineages is controlled by miR-204 through suppressing of Runx2 and DVL3 expression [229]. Preadipocyte differentiation can be both inhibited (e.g., by miR-125a-5p, which negatively regulates STAT-3 [230]) and enhanced (e.g., by miR-183 which targets LRP6 [231]) by microRNAs. Many studies highlight the importance of microRNAs in the regulation of brown, brite and white adipose tissue differentiation [228]. Moreover, in mice with adipose tissue specific DICER (a key protein in microRNA biogenesis) knockout whitening of brown adipose tissue was observed [232]. In addition to the single examples of microRNAs involved in adipocytogenesis are given above, there are several dozen other microRNAs in the literature, the expression level of which depends on the degree of differentiation of fat cells [233]. The expression of microRNAs, as well as the expression of genes encoding proteins, must therefore be controlled. One of the factors regulating the level of microRNA expression are gonadal steroids [33,34,35,36,37]. As shown in Table 1, the level of several microRNAs important in the development and differentiation of adipose tissue can be regulated by sex hormones.

The function of microRNA in fat cells is not limited to controlling cell differentiation. Numerous works document the influence of microRNAs on the metabolism and function of fat cells [228]. For example, the level of UCP1 protein that modulates glucose metabolism and heat production has been shown to be regulated by miR-191a-5p, miR-204 and miR-455 [260,261,262]. The level of GLUT4, the protein responsible for glucose uptake in BAT cells, is regulated directly by miR-93 and miR-218 [263,264]. A correlation between the expression level of miR-222, the estrogen receptor and GLUT4 has also been demonstrated [265]. microRNAs which expression can be regulated by androgens (miR-124 and miR-145) and estrogens (miR-30c) are implicated in thermogenesis regulation by targeting MCT1, LDH and RIP140, respectively [266,267,268]. MCT1 expression is also modulated by miR-342-3p [269]. On the other hand, miR-124 is one of the modulators of fat metabolism by inhibiting TLG expression [270]. Another androgen-regulated microRNA (miR-128) is involved in lipid accumulation via repressing SIRT1 expression [271]. In WAT cells, progesterone-regulated miR-193b controls adiponectin production [272].

Apart from maintaining the proper energy balance of the body, adipose tissue also performs a secretory function. Autocrine, paracrine or endocrine communication in adipose tissue occurs via exosomes—vesicles produced and released, among others, by adipocytes [273]. Exosomes participate in intercellular and interorgan communication by transporting bioactive molecules produced in fat cells to their target cells. One type of active molecule transported by exosomes are microRNAs. Studies on mice with a knockout of the DICER protein coding gene and on patients with lipodystrophy have shown that fat cells (especially BAT) are the major source of circulating exosomal microRNAs [15]. Exosomes secreted by adipose tissue can be identified in biological fluids by measuring adipocyte-enriched microRNAs (let-7b, miR-16, miR-103, miR-146b, miR-148a, miR-201, miR-221 and miR-222) [274]. Although a direct relationship between sex hormones and microRNAs expressed in adipocytes has not been established in adipocyte cells, the expression of most of them can be regulated by gonadal steroids [34,35,36,37]. There is evidence that adipocyte-derived extracellular vehicles can influence the function of liver, skeletal muscle, heart, lungs, hypothalamus and ovary [274,275]. The composition and content of exosomes strongly depend on the physiological state of the body and there is increasing evidence that adipose-derived vehicles play an important role in the dysregulation of the body’s energy balance and metabolism [273]. Knowledge on the role of miRNAs in the regulation of metabolism and development of metabolic diseases is developing intensively [276]. The role of miRNAs in the development of metabolic diseases such as obesity, T2D, polycystic ovary syndrome, nonalcoholic fatty disease, cardiovascular and inflammatory diseases was recently reviewed [277,278,279,280,281,282,283]. Moreover, the expression of aquaporin isoforms associated with metabolic diseases is also regulated by microRNAs [284]. Studies describing the regulation of microRNAs expression by gonadal steroids [34,35,36,37] and the association of microRNAs with metabolic diseases [277,278,279,280,281,283,284,285], allowed to identify the most important microRNAs in metabolic diseases which expression level may be regulated by sex hormones (Table 2).

## 6. Discussion

Several aspects of adipose tissue biology are regulated differently in males and females, influencing their predisposition to metabolic disorders. Moreover, disturbances in gonadal hormone homeostasis can deeply affect energy balance, glucose and lipid metabolism, reinforcing the need for personalized sex-specific approaches in management of metabolic abnormalities. This kind of approach should take into account the possibilities of regulation of gonadal steroids action at adipose tissue level. Below, we summarize the most promising, relatively novel therapeutic tactics fitting into the trend of personalized management of metabolic disorders. We also place a great emphasis here on these drug targets which are still uncommonly considered in the field.

Aquaporins are emerging drug targets to prevent and counteract metabolic abnormalities [43,286]. Among the substances that could improve adipocyte functioning in both sexes are thiazolidinediones (being insulin sensitizers) acting as PPARγ agonists, which are proven to upregulate AQP7 [43,199,200,287].

Considering the effects of gonadal hormones on the ability of the organism to respond to insulin, it is reasonable to consider the use of insulin sensitizers to alleviate the steroid-tuned insulin resistance. One of them is metformin which is commonly used in T2D [288]. As mentioned in Section *Insulin sensitivity*, estradiol is able to enhance the activity of adenosine monophosphate-activated protein kinase (AMPK) through ER [121]. In turn, one of the molecular mechanisms of metformin action includes AMPK activation [288]. From this perspective, metformin administration seems a reasonable choice in case of prevention of insulin resistance especially in patients with E2 deficits.

Other promising substances are inositols, which not only improve insulin sensitivity but also regulate steroidogenesis in women, and consequently may refine ovulatory function in hyperandrogenic females [289,290]. Interestingly, the use of myoinositol improves sperm function, boosting sperm motility in patients with altered semen parameters (in asthenozoospermia) [291]. It is worth noticing that the sperm motility and volume, affecting the observed fertility, are regulated by aquaporins [292]. In connection with the above, male patients with idiopathic infertility could also take advantage of inositol supplementation. Thus, inositols may find clinical applications in assisted reproductive treatment and counteracting metabolic dysfunctions in both men and women [293].

Other active substances that can be used to improve endocrine function of adipocytes in cases of corticosteroid imbalance are those affecting the immune system. Considering the significant role of adiponectin in counteracting inflammatory processes and increasing the sensitivity of tissues to insulin, this adipokine may be considered as a potential drug [147,290]. Exogenous adiponectin supplementation in early pregnant individuals may help to avoid the metabolic syndrome of adult female offspring, according to the recent studies on a mouse PCOS model [294]. A probable mechanism underlying this effect is related to the activation of the AMPK/PI3K-Akt signaling pathway in PCOS offspring mice [294].

Since AQPs have been recognized as functionally involved in immune cell activity such as priming and inflammasome activation, transendothelial migration and phagocytosis, development of AQP-oriented drugs gives a promising opportunity in future therapies against metabolic diseases [203]. Up to date, several AQP modulators have been proposed and even patented for use for diagnostic and therapeutic purposes [295,296]. Nevertheless, the lack of selectivity and toxic side effects prevent most of these substances from being subjected to extensive clinical testing. Among the most promising AQP modulators, which are relatively safe and able to effectively prevent inflammation, are the gold(III) bipyridyl compound Auphen and compounds DFP00173, Z433927330 and HTS13286. The physiological effects of their administration are summarized in [203]. An alternative AQPs-targeted approach can be based on modulation of aquaporins at the post-transcriptional level.

A global regulator of gene expression are microRNAs. Although sex-related differences in the expression profile of microRNAs are still poorly understood [282], there is evidence that both sex chromosomes and sex hormones can cause differences in microRNAs expression [33]. At the same time, microRNAs can be a link between coexisting metabolic diseases, as each microRNA can interact with dozens of targets. Thus, microRNAs represent promising therapeutic target itself. Our analysis (Table 2) suggests that miR-29 may be one of the therapeutic targets, as its deregulation is associated with all analyzed metabolic disorders. Equally interesting, in the context of the role of adipose tissue in the development of metabolic diseases, are adipocyte-enriched microRNAs (let-7b, miR-16, miR-146b, miR-221 and miR-222) associated with metabolic disorders.

Recently, significant attention has been drawn to application of flavonoids to attenuate chronic inflammation [297,298]. It has been reported that natural flavonoids (e.g., naringenin, rutin and quercetin) can influence modulatory effects on inflammasomes associated with the initiation and progression of chronic disorders, including metabolic ones. Regardless of sex, inflammasome targeting via flavonoids can bring advantageous effects on health. As an example, naringenin increases the concentration of enzymes removing reactive oxygen species and exhibits cytoprotective and anti-inflammatory effects via targeting mitochondrial potassium channels [299]. Naringenin can also modulate the activity of enzymes involved in interconversion of steroid hormones (3β-hydroxysteroid dehydrogenase and 17β-hydroxysteroid dehydrogenase), as summarized in [290]. Thus, it can be useful in downregulation of androgen production in women suffering hyperandrogenemia.

Other possible strategy to improve functioning of adipose tissue in metabolic diseases is to target adipocyte mitochondria, due to their substantial roles in the regulation of whole-body energy homeostasis, control of insulin sensitivity and glucose metabolism or crosstalk between muscles and adipose tissues [300]. Here, possible therapeutic solutions include the activation of BAT thermogenesis and WAT browning [301] and the application of mitochondrial-targeted antioxidants (such as vitamin E, N-acetylcysteine, glutathione and coenzyme Q10). It is worth noticing that WAT browning may be induced by the chronic treatment with β3-adrenergic activators or the PPARγ agonist thiazolidinedione [300], which was also reported in this review as an AQP-oriented drug. In turn, affecting the adaptive thermogenesis in brown adipose tissue can also be achieved by the administration of β-adrenergic agonists affecting sympathetic nervous system and promoting increased BAT activity [301]. Women have more active brown adipose tissue (BAT) than men [302], and this relation may be partly caused by the differences in sex hormone levels. Estradiol can activate thermogenesis in BAT (promoting energy expenditure) through the sympathetic nervous system (SNS) due to its ability to inhibit AMPK through α-ER selectively in the ventromedial nucleus of the hypothalamus (VMH) [302,303]. Even the physiological changes in E2 levels across the menstrual cycle affect thermogenesis in women. Thus, the disruption of the female hormonal balance impairs the energy homeostasis also at the level of VMH AMPK-SNS-BAT axis which may contribute to the tendency to obesity in women with estrogen deficits [303]. Moreover, both rodent models as well as the characteristics of the PCOS patients suggest that BAT thermogenesis is negatively associated with androgen levels [304,305].

Coming back to the possibilities of sex-dependent therapies of metabolic diseases, one may consider the potential procedures dedicated to modulate the activity or levels of steroid-converting enzymes in human adipose tissues. With the proceeding fat accumulation in obese men, there is an increase of aromatase activity that is associated with a greater conversion of testosterone to estradiol (Figure 2). Thus, the resulting depression of testosterone concentrations give rise to the increased preferential deposition of abdominal fat, which deepens the hypogonadal state [306]. Another enzyme that can serve as a target in therapies against metabolic abnormalities, is type 12 17β-HSD, which is reported as mainly responsible for the conversion of estrone into estradiol according to the in vitro studies [307] (Figure 2). The regulation of expression level of this enzyme, which is maximally expressed at the end of the differentiation in human adipocytes, could represent a mechanism that allows a time-dependent and cell type-specific tuning of estrogen production [307].

The eventual solution to the metabolic complications accompanied by sex hormone imbalance seems to be offered by the introduction of hormone replacement therapies. The administration of gonadal hormones could be considered in the groups of elderly people, patients with morbid obesity, women suffering estrogen depletion (e.g., postmenopausal women) and men exhibiting androgen deficits (hypogonadal men). According to the literature [68,308], estradiol supplementation seems to be efficient in prevention of metabolic consequences of the long-term estrogen deficiency (as in the case of ovariectomy or postmenopausal women). Estradiol is one of the factors regulating energy homeostasis by modulating both energy expenditure (modulation of BAT thermogenesis) and food intake. E2 can inhibit feeding while acting in arcuate nucleus of the hypothalamus, whereas estrogen deprivation is related to hyperphagia and, frequently, weight gain [309,310]. According to the ovariectomized mice model, these effects can be reversed by E2 replacement therapy [309]. Moreover, as suggested by a mouse model [311], supplementation of individuals exhibiting reduced ovarian functions with estrogen can revert such effects, such as increased 5${}^{\prime }$adenosine monophosphate-activated protein kinase (AMPK) phosphorylation, high expression level of the genes encoding adiponectin, UCP2 and PPARγ coactivator 1α (PGC-1α) and low resistin expression in WAT. Additionally, estradiol treatment enables attenuating the decrease of perilipin in VAT after bilateral ovariectomy [308], which is an important outcome, since perilipin is the principal protein controlling the lipase access to stored triglycerides and consequently regulating lipolysis [308]. To sum up, estradiol replacement seems to be a promising therapeutic solution for women suffering E2 depletion. However, introduction of this kind of therapy should be highly personalized and take into account the possible side effects of chronic hormone supplementation.

The testosterone replacement therapy (TRT) is particularly advantageous in hypogonadal men, where its application results in enhanced energy metabolism, increased fat-free mass, decreased level of inflammatory markers and improved sexual function [155,312,313,314]. The improvement of patients’ health is especially evident in hypogonadal men with T2D [315], for whom testosterone replacement therapy for 1 year or longer allows increasing their survival probability. Importantly, beside the benefits of TRT therapy in this group of patients at the metabolic and sexual levels, it is also advantageous in case of infections (e.g., viral diseases) [175] due to the possible mitigation of the damaging inflammatory response to a given pathogen without hampering the immune system’s response.

As a general comment, it is clear from the literature that, while the popular rodent models admittedly give interesting inferences, they are not ideal when considering the subtle dynamics of hormone-regulated processes. In this review, we only mention some, subjectively chosen, reports on mice or rat models, only when they highlight the most important aspects of the discussed processes. The results of human studies often contrast those obtained in animals, which may resemble the significance of species-inherent details of hormonal balance. To explain these discrepancies, extensive in vitro studies on molecular mechanisms are paramount. From this perspective, at the initial stages of research on human physiology of sex hormone-regulated processes, it seems that the best choice is to carry out experiments in vitro on human cells, if it is possible.

## 7. Conclusions

The well-known guidelines to prevent and alleviate metabolic disorders include a set of lifestyle choices referring to dietary habits, exercise and caloric restriction. Their impact is, however, far below expectations in the general population. To reach satisfactory results in counteracting versatile abnormalities in energy, glucose and lipid homeostasis, novel therapeutic strategies should be proposed. This is where personalized medicine procedures can be particularly useful. According to the complex mechanisms responsible for sex-dimorphic regulation of adipose tissue functioning, the novel therapeutic solutions should take into account the sex of the patient and the current status in their hormonal balance. In such strategy, not only should the conventional biochemical agents be considered as molecular targets but also the more fundamental ones such as miRNAs. On the other hand, aquaporins emerge as novel biologic targets in sex-specific management of metabolic diseases. Recognition of active substances affecting these specific targets offers a promise for the management of a large spectrum of clinical disorders including metabolic and energy balance diseases.

## Figures and Tables

**Table 1:** Gonadal steroid-regulated microRNAs involved in adipogenic differentiation.

miRNA	Regulation	Study Model	References
let-7a	A; E	3T3-L1	[234]
let-7c	A; E	3T3-L1	[234]
miR-9	E	3T3-L1	[235]
miR-17-5p	A; E; P	3T3-L1	[236]
miR-21	A; E; P	BMSCs	[237]
miR-22	A	knockout mice; primary brown adipocytes	[238]
miR-23b	A; E; P	knockout mice; primary pre-adipocytes	[239]
miR-24	E	3T3-L1	[240]
miR-26	E; P	C57Bl/6 mice; SVF	[241]
miR-27a	A; E	mature adipocytes; SVCs; MSC	[242]
miR-29	A; E	hMADS	[243]
miR-30d	A; E	hMADS	[244]
miR-32	A	MCPIP1; 3T3-L1	[245]
miR-124	A	hMSC	[246]
miR-128	A	hMSC	[247]
miR-129	E	C57BLKS/J mouse	[248]
miR-133	A	3T3-L1	[249]
miR-143	E	3T3-L1	[250]
miR-144	E	3T3-L1; mice	[251]
miR-150	E	pre-adipocytes from Qinchuan cattle	[252]
miR-182	A; E	3T3-L1; VAT	[253]
miR-203	A; E	Knockout mice; SVF	[84]
miR-204	A	3T3-L1	[254]
miR-206	E	3T3-L1	[255]
miR-221	A	SGBS	[256]
miR-342	E	hMSC; 3T3-L1	[257]
miR-363	A	ADSCs	[231]
miR-375	A; E	3T3-L1	[258]
miR-378	E	Bovine preadipocytes	[259]

Abbreviations: A, androgens; E, estrogens; P, progesterone. Table 1 is based on [34,35,36,37] (Regulation) and references given in the table (miRNA, Metabolic Disorders).

**Table 2:** Gonadal steroid-regulated microRNAs involved in metabolic diseases and AQPs functioning.

miRNA	Regulation	Metabolic Disoders	References
let-7a	E	PCOS	[279]
let-7b	E	O, T2Ds, PCOS	[277,278,279]
let-7c	A, E	O, T2Ds, PCOS	[277,278,279]
miR-1	A	T2D, AQP	[278,284]
miR-9	E	PCOS, NAFLD	[279,280]
miR-15	E	PCOS, NAFLD, CVD	[279,280,282]
miR-16	E	PCOS, NAFLD, AQP	[279,280,284]
miR-19	A	PCOS, NAFLD, AQP, CVD	[279,280,282,284]
miR-20	P	T2D	[278]
miR-21	A, E, P	PCOS, NAFLD, AQP	[279,280,284]
miR-22	A	O, AQP	[277,284]
miR-23a	P	PCOS, AQP, CVD	[279,282,284]
miR-24	E	PCOS, NAFLD, CVD	[279,280,282]
miR-26a	A	O, PCOS, NAFLD	[277,279,280]
miR-27	A, E	PCOS, NAFLD, CVD	[279,280,282]
miR-29	A, E, P	O, T2D, PCOS, NAFLD, AQP	[277,278,279,280,284]
miR-30d	A, E	T2D, PCOS, NAFLD	[278,279,280]
miR-32	A	PCOS, AQP, CVD	[279,282,284]
miR-92	E	PCOS	[279]
miR-99	A	PCOS, NAFLD	[279,280]
miR-100	A	PCOS	[279]
miR-124a	A	T2D, PCOS, AQP	[278,279,284]
miR-125	A, E	PCOS, NAFLD, CVD	[279,280,282]
miR-128	A	PCOS	[279]
miR-133a	A	T2D, PCOS	[278,279]
miR-135	A, E	PCOS	[279]
miR-141	A, E	PCOS	[277,279]
miR-142	P	O, PCOS, CVD	[277,279,282]
miR-143	E	O	[277]
miR-144	E	O, PCOS, NAFLD, AQP	[277,279,280,284]
miR-145	A	T2D, AQP	[278,284]
miR-146	P	PCOS, NAFLD	[279,280]
miR-148	A	O	[277]
miR-149	E	PCOS, NAFLD	[279,280]
miR-151	E	PCOS	[279]
miR-182	A, E	PCOS, NAFLD, CVD	[279,280,282]
miR-185	A	PCOS, AQP	[279,284]
miR-193	P	PCOS	[279]
miR-195	E	PCOS, AQP	[279,284]
miR-200	P	T2D, PCOS, NAFLD	[278,279,280]
miR-203	A, E	PCOS, NAFLD, AQP	[279,280,284]
miR-221	A	O, PCOS, NAFLD, CVD	[277,279,280,282]
miR-222	A	O, PCOS, NAFLD, CVD	[277,279,280,282]
miR-320	E	PCOS, AQP	[279,284]
miR-342	E	PCOS	[279]
miR-365	E	PCOS	[279]
miR-375	A, E	O, T2D	[277,278]
miR-423	A, E	O	[277]
miR-432	E	O	[277]
miR-486	E	PCOS	[279]
miR-504	E	O	[277]
miR-520	E	O	[277]
miR-548	E	O	[277]
miR-690	A	T2D	[278]

Abbreviations: A, androgens; E, estrogens; P, progesterone; O, obesity; T2D, type 2 diabetes; PCOS, polycystic ovary syndrome; NAFLD, non-alcoholic fatty liver disease; AQP, aquaporins; CVD, cardiovascular diseases. Table 2 is based on [34,35,36,37] (Regulation) and the other references are given in the table (miRNA, Metabolic Disorders).

## Abbreviations

The following abbreviations are used in this manuscript:

ACC1	acetyl-CoA carboxylase 1
AKR1C	aldo–keto reductase 1C enzyme
AKT	protein kinase B
AMPK	5${}^{\prime }$AMP-activated protein kinase
AR	adrenergic receptors
ATGL	adipose triglyceridelipase
BAT	brown adipose tissue
3β-HSD	3β-hydroxysteroid dehydrogenase
11β-HSD1	11β-hydroxysteroid dehydrogenase type 1
17β-HSD	17β-hydroxysteroid dehydrogenase
CD68	cluster of differentiation 68
CRP	C-reactive protein
CYP11A1	cholesterol side-chain cleavage enzyme
CYP17A1	steroid 17α-monooxygenase
DHEA	dehydroepiandrosterone
DHEA-S	dehydroepiandrosterone sulfate
DHT	dihydrotestosterone
E1	estrone
E2	17β estradiol
E3	estriol
FAS	fatty acid synthase
FFA	free fatty acids
GLUT4	glucose transporter type 4
IRE	insulin response element
IRS-1	insulin receptor substrate 1
HSL	hormone-sensitive lipase
LPL	lipoprotein lipase activity
NAFLD	non-alcoholic fatty liver disease
NF-κB	nuclear factor kappa-light-chain-enhancer of activated B cells
PBMCs	peripheral blood mononuclear cells
PCOS	polycystic ovary syndrome
PKCζ	protein kinase C ζ
PPARγ	peroxisome proliferator-activated receptor γ
SAT	subcutaneous adipose tissue
SCD1	stearoyl-CoA desaturase 1
SULT2A1	dehydroepiandrosterone sulfotransferase
T	testosterone
T2D	diabetes mellitus type 2
TLR	Toll–Like Receptors
TNFα	tumor necrosis factor
TRT	testosterone replacement therapy
VAT	visceral adipose tissue
WAT	White adipose tissue
